# Real-World Prevalence and Outcomes of Patients with Paroxysmal Nocturnal Hemoglobinuria Treated with C5 Inhibitors in the US: A Retrospective Claims Database Analysis

**DOI:** 10.36469/001c.142049

**Published:** 2025-08-15

**Authors:** Srinivas K Tantravahi, Dominick Latremouille-Viau, Raj Desai, Soyon Lee, Jincy Paulose, Anumaxine Geevarghese, Annie Guérin, Shravanthi Seshasayee, Mohin Chanpura, Glorian Yen

**Affiliations:** 1 Huntsman Cancer Institute, University of Utah, Salt Lake City, Utah; 2 Groupe d’analyse, Ltée, Montréal, QC, Canada; 3 Analysis Group, Inc, Boston, Massachusetts; 4 Novartis Pharmaceuticals Corp., East Hanover, New Jersey; 5 Groupe d’analyse, Ltée, Montréal, Québec, Canada; 6 Novartis Pharmaceuticals Corp., East Hanover, NJ, USA

**Keywords:** paroxysmal nocturnal hemoglobinuria, eculizumab, ravulizumab, healthcare resource utilization, direct healthcare costs, breakthrough hemolysis, blood transfusion, thrombosis

## Abstract

**Background:**

Paroxysmal nocturnal hemoglobinuria (PNH) is a rare blood disorder with C5 inhibitors (C5i), eculizumab and ravulizumab, being part of current treatment options.

**Objectives:**

To estimate the 5-year prevalence of PNH and describe the healthcare resource utilization and direct healthcare costs associated with C5i among commercially insured patients with PNH treated with C5i in the US.

**Methods:**

The 5-year prevalence of adults with PNH in IQVIA PharMetrics® Plus was estimated (2018-2022). A retrospective cohort study (2011-2022) was also conducted in adults with PNH treated with C5i and ≥3 months of continuous health plan coverage following the first claim for C5i (index date). PNH-related health resource utilization and direct healthcare costs were assessed from index date until earliest of treatment discontinuation/end of data/end of continuous health plan coverage (follow-up period).

**Results:**

The 5-year prevalence of PNH was 2.4 per 100 000 persons in commercial claims. A total of 371 patients treated with C5i (median age: 40 years; female: 55.3%; eculizumab: 53.9%; ravulizumab: 46.1%) were followed for a mean ± SD [median] of 19.3 ± 16.9 [14.7] months. Annual incidence rates of PNH-related blood transfusion and breakthrough hemolysis (BTH) among patients treated with C5i were 1.2 (eculizumab: 1.3; ravulizumab: 1.0) and 4.5 (eculizumab: 5.2; ravulizumab: 3.3) per person per year (PPPY), respectively. In patients treated with eculizumab and ravulizumab, respectively, PNH-related blood transfusion was required by 46.2% and 11.9% of patients in the first 6 months post-index, and over the follow-up period, transfusion avoidance was observed in 46.2% and 78.2% of patients. The 6- and 12-month rates of PNH-related thrombosis were 8.0% and 10.6% for eculizumab and 6.1% and 11.6% for ravulizumab, respectively. Among patients treated with C5i, estimated annual total PNH-related costs PPPY were 660 533(eculizumab:697 459; ravulizumab: 612 522)forthefirstyearand633 984 (eculizumab: 691 022;ravulizumab:570 832) for subsequent years, with treatment costs accounting for 94.3% to 94.6% of total costs.

**Discussion:**

Despite treatment with C5i, patients with PNH still exhibited BTH, required blood transfusions, and experienced thrombosis.

**Conclusion:**

This study highlights the unmet need for more effective PNH treatments to address the economic and clinical burden associated with PNH and improve disease control among patients.

## BACKGROUND

Paroxysmal nocturnal hemoglobinuria (PNH) is a hematologic disorder characterized by complement-mediated hemolysis that can cause subsequent life-threatening anemia and thrombosis. One or more acquired mutations in the phosphatidylinositol glycan anchor biosynthesis class A (*PIGA*) gene lead to the loss of the complement regulatory proteins CD55 and CD59 from the surface of red blood cells, making them vulnerable for degradation by the membrane attack complex. PNH is a rare disease, affecting an estimated 1.3 per 100 000 people in the United States. As of 2020, treatments for patients with PNH included the C5 inhibitors eculizumab and ravulizumab, which inhibit formation of the complement-initiated membrane attack complex and consequently prevent intravascular hemolysis. More recently, iptacopan, an oral complement factor B inhibitor; danicopan, an add-on therapy for C5 inhibitors; pegcetacoplan, a C3 inhibitor; and crovalimab, another C5 inhibitor, have also received US Food and Drug Administration (FDA) approval for the treatment of PNH. As patients with PNH require long-term monitoring and management, the condition can be associated with considerable healthcare resource utilization (HRU) and direct healthcare costs, regardless of the treatment status, with approximately $18 978 in average costs per patient per month (PPPM). Furthermore, in a study evaluating these costs among patients treated with C5 inhibitors, Broderick et al found that, on average, the total annual healthcare costs associated with eculizumab and ravulizumab were estimated to be $711 785 and $624 911, respectively. Although C5 inhibitors can effectively alleviate intravascular hemolysis, they do not affect extravascular hemolysis mediated by the complement protein C3d, resulting in incomplete response and persistent symptoms in some patients with PNH. In addition, breakthrough hemolysis (BTH) can still occur with C5 inhibitor treatment, which may necessitate blood transfusions or additional C5 inhibitor doses to re-establish disease control. In the study by Broderick et al, 29.4% and 13.5% of patients treated with eculizumab and ravulizumab, respectively, experienced BTH, while 14.7% and 13.5%, respectively, experienced thrombosis. In a separate study of patients with PNH treated with eculizumab, Cheng et al found that patients who were blood transfusion dependent during treatment incurred higher costs than patients who were blood transfusion free, which was driven primarily by an increased rate of hospitalizations, underscoring the potential severity and consequence of these events.

Despite the clinical challenges experienced by patients with PNH receiving treatment with C5 inhibitors, limited real-world evidence on HRU and healthcare costs exists in the US outside of these two studies, with limited sample size. In light of this, there is a need for a more comprehensive understanding of the economic and clinical burden of patients with PNH receiving treatment with C5 inhibitors, who may continue to experience ongoing disease activity, using more recent real-world data and larger sample sizes. To that end, this study aimed to estimate the contemporary prevalence of PNH and describe HRU and direct healthcare costs associated with C5 inhibitors among patients with PNH treated with eculizumab or ravulizumab in US clinical practice using a large commercial health plan data source.

## METHODS

### Data Source

This study used a large US health insurance claims database, IQVIA PharMetrics® Plus, and included data from January 1, 2011, to September 30, 2022 (study period). The database is composed of fully adjudicated, integrated medical and pharmacy claims data for over 215 million unique enrollees. It is representative of the commercially insured US population for patients aged under 65 years and contains a longitudinal view of inpatient and outpatient services, prescription and office/outpatient-administered drugs, provider specialty, costs (including allowed and paid amounts), patient demographics (including state), and detailed enrollment information. As all data were de-identified and compliant with the Health Insurance Portability and Accountability Act, no institutional review board approval was required. At the time the study was conducted, the health plan claims did not include iptacopan, danicopan, or crovalimab; availability of pegcetacoplan was limited.

### Study Design

A retrospective cohort study using data from IQVIA PharMetrics® Plus database was conducted. Data from 2018 to 2022 were used to assess the prevalence of PNH in commercially insured adult patients.

Furthermore, data from 2011 to 2022 were used to assess study outcomes (as described below). We included adult patients having at least 1 claim with a primary or secondary diagnosis of PNH (*International Classification of Diseases, Tenth Revision, Clinical Modification* [ICD-10-CM] diagnosis: D59.5) in any setting (inpatient or outpatient) and treated with a C5 inhibitor (eculizumab [Healthcare Common Procedure Coding System (HCPCS): J1300, C9236; *International Classification of Diseases, Tenth Revision, Procedure Coding System* (ICD-10-PCS): XW033C6, XW043C6; National Drug Codes (NDC) from Generic Product Identifier (GPI): 8580505000] or ravulizumab [HCPCS: J1303, C9052; NDC from GPI: 8580508020]) during the study period.

The index date was defined as the first claim for either eculizumab or ravulizumab and patients were required to have at least 3 months of continuous health plan enrollment post-index. Patients were excluded if they had another condition treated with C5 inhibitors (ie, neuromyelitis optica spectrum disorder, generalized myasthenia gravis, or atypical hemolytic uremic syndrome), had codes indicating participation in a clinical trial, or had previously received a hematopoietic stem cell transplantation.

The follow-up period started from the index date until the earliest of treatment discontinuation (treatment gap of more than 42 days for eculizumab, or more than 112 days for ravulizumab), the end of continuous health plan enrollment, or the end of data availability (Sept. 30, 2022). Among patients with at least 6 months of continuous health plan enrollment pre-index, the induction phase was defined as the first 28 days following the index date for patients treated with eculizumab, or the first 14 days following the index date for patients treated with ravulizumab. Among all patients meeting the eligibility criteria, the maintenance phase spanned from day 29 or day 15 post-index for patients treated with eculizumab or ravulizumab, respectively, until the end of the follow-up period.

### Study Outcomes

PNH-related HRU (based on diagnosis of PNH or procedure for C5 inhibitor codes) was measured during the follow-up period for inpatient admissions, emergency department (ED) visits, and days with outpatient services. PNH-related blood transfusions and BTH were also assessed, with PNH-related blood transfusions defined as a day with a medical claim for a blood transfusion procedure(s) in any setting (inpatient, ED, outpatient), along with a diagnosis for PNH.

BTH-related events in any setting were based on either BTH symptoms or treatments. BTH-related events based on symptoms of BTH were defined as at least 1 of the following conditions: abdominal pain, anemia (excluding aplastic anemia), chronic kidney disease, dysphagia, dyspnea, erectile dysfunction, fatigue, infection, sepsis, respiratory infection, kidney infection, urinary tract infection, pulmonary hypertension, or thrombosis (**Supplemental Table S1**), along with a diagnosis of PNH. BTH-related events based on treatments for BTH were defined as a PNH-related blood transfusion, characterized by a medical service with a diagnosis of PNH and a procedure for blood transfusion, or a change in C5 inhibitor dosing schedule (ie, shorter interval or dose escalation). Among the subgroup of patients observed from induction, PNH-related blood transfusion, transfusion avoidance (TA), and PNH-related thrombosis were measured during the follow-up period. Among patients with at least 6 months of follow-up, TA was defined as receiving no blood transfusions from the index date to the end of the follow-up period. PNH-related thrombosis was defined as at least 1 of the following conditions: venous thrombosis (including portal vein thrombosis [Budd-Chiari syndrome]), pulmonary embolism, pulmonary hypertension, arterial thrombosis, or cerebral venous sinus thrombosis (**Supplemental Table S1**), along with a diagnosis of PNH.

PNH-related direct healthcare costs, including total costs, medical costs in the inpatient, ED, and outpatient settings, and pharmacy costs, were measured during the follow-up period. Costs of blood transfusion and BTH at the event level were also evaluated. All costs were estimated from the payers’ perspective, adjusted for inflation using the Consumer Price Index, and reported in 2022 US dollars. No inflation adjustment was performed for C5 inhibitor treatment costs.

### Statistical Analyses

The 5-year prevalence of PNH was calculated as the number of adult patients with PNH (at least 1 medical claim with ICD-10-CM diagnosis: D59.5) with health plan enrollment between 2018 and 2022 divided by the total number of adult patients with health plan enrollment during the same period.

Descriptive analyses of PNH-related HRU and associated direct healthcare costs were reported separately for patients treated with C5 inhibitors, eculizumab, and ravulizumab. The C5 inhibitor cohort included the most recent agent. PNH-related HRU was reported as the annual incidence rate (IR) per patient per year (PPPY), while PNH-related direct healthcare costs were reported in the induction phase and PPPM in the maintenance phase, separately. Time to PNH-related thrombosis was measured from the index date to the first medical event of PNH-related thrombosis or censored at the end of the follow-up period and assessed using Kaplan-Meier (KM) analysis.

Annualized total PNH-related costs PPPY were estimated over the first year of treatment as the mean total PNH-related cost in the induction phase plus the mean total PNH-related cost PPPM in the maintenance phase multiplied over 11 months. For subsequent treatment years, the annualized PNH-related costs PPPY were calculated as the mean total PNH-related costs PPPM during the maintenance phase multiplied over 12 months. Data management and analyses were performed using SAS Enterprise Guide software version 7.1 (SAS Institute, Cary, North Carolina).

## RESULTS

### Prevalence of PNH in Commercially Insured Patients

Between 2018 and 2022, the 5-year prevalence of PNH among commercially insured patients was 2.4 cases per 100 000 persons and the average annual compound growth rate was 3.2%. Over the same period, 30.0% of patients were observed to have at least 1 claim for a complement inhibitor (eculizumab: 16.4%; ravulizumab: 21.9%; pegcetacoplan: 2.2%).

### Patient Characteristics

From 2011 to 2022, a total of 371 patients met the study eligibility criteria and received treatment for PNH with a C5 inhibitor (induction phase: n = 175; maintenance phase: n = 362) (**Supplemental Figure S1**). Overall, 288 patients were treated with eculizumab (induction phase: n = 83; maintenance phase: n = 278) and 171 patients were treated with ravulizumab (induction phase: n = 117; maintenance phase: n = 171). Among patients treated with C5 inhibitors, the mean ± SD age at PNH diagnosis was 41.3 ± 13.8 years and 55.3% of patients were female (**Table 1**). Patients treated with eculizumab and ravulizumab had a mean ± SD age at PNH diagnosis of 41.9 ± 13.6 years and 39.1 ± 12.2 years, respectively. Among those who received eculizumab and ravulizumab, 60.4% and 42.7% were female, respectively. The mean ± SD (median) follow-up duration was 19.3 ± 16.9 (14.7) months for patients treated with a C5 inhibitor, 23.2 ± 22.4 (15.0) months for those treated with eculizumab, and 17.0 ± 10.8 (14.5) months for patients treated with ravulizumab.

**Table 1.** Patient Demographic and Clinical Characteristics

	**Any C5 Inhibitor Treatment** **(n = 371)**	**Eculizumab Induction Phase (n = 83)**	**Eculizumab Maintenance Phase (n = 278)**	**Ravulizumab Induction Phase (n = 117)**	**Ravulizumab Maintenance Phase (n = 171)**
Demographic characteristics^a^					
Age at index date,^b^ mean ± SD [median]	41.3 ± 13.8 [40.0]	42.7 ± 14.7 [42.0]	41.9 ± 13.2 [41.0]	40.5 ± 12.1 [40.0]	39.1 ± 12.2 [39.0]
Female, n (%)	208 (55.2)	50 (60.2)	168 (60.4)	53 (45.3)	73 (42.7)
US Census region, n (%)					
Northeast	48 (12.7)	10 (12.0)	37 (13.3)	15 (12.8)	21 (12.3)
South	182 (48.3)	32 (38.6)	135 (48.6)	48 (41.0)	80 (46.8)
Midwest	97 (25.7)	32 (38.6)	68 (24.5)	43 (36.8)	53 (31.0)
West	49 (13.0)	8 (9.6)	37 (13.3)	11 (9.4)	17 (9.9)
Commercial insurance type, n (%)					
Consumer-directed healthcare	6 (1.6)	2 (2.4)	5 (1.8)	0 (0.0)	1 (0.6)
HMO	57 (15.1)	11 (13.3)	39 (14.0)	13 (11.1)	21 (12.3)
Indemnity/traditional	1 (0.3)	0 (0.0)	1 (0.4)	0 (0.0)	1 (0.6)
Point of service	27 (7.2)	9 (10.8)	20 (7.2)	11 (9.4)	14 (8.2)
PPO	286 (75.9)	61 (73.5)	213 (76.6)	93 (79.5)	134 (78.4)

Abbreviations: HMO, health maintenance organization; PPO, preferred provider organization.

^a^Patient demographics were summarized as of the index date.

^b^Index date was defined as the first claim (medical or pharmacy claim) for eculizumab or ravulizumab. Ravulizumab received US Food and Drug Administration approval in December 2018.

### PNH-Related HRU

The annual IR of PNH-related inpatient admissions was 0.2 PPPY among patients treated with C5 inhibitors, 0.2 PPPY among patients treated with eculizumab, and 0.1 PPPY among patients treated with ravulizumab (**Table 2**). Patients treated with C5 inhibitors had an annual IR of PNH-related blood transfusion of 1.2 PPPY, while BTH-related events in any setting ranged from 3.2 PPPY to 4.5 PPPY depending on whether BTH was defined by symptoms of or treatments for BTH. Patients treated with eculizumab and ravulizumab had an annual IR of 1.3 PPPY and 1.0 PPPY for PNH-related blood transfusion, respectively, and BTH-related events in any setting ranged from 4.8 PPPY to 5.2 PPPY among patients treated with eculizumab and 1.2 PPPY to 3.3 PPPY among patients treated with ravulizumab, depending on the BTH definition used.

**Table 2.** PNH-Related HRU in Patients With PNH Treated With a C5 Inhibitor

**Annual Incidence Rate, PPPY**	**Any C5 Inhibitor Treatment (n = 371)**	**Eculizumab (n = 288)**	**Ravulizumab (n = 171)**
Inpatient admissions	0.2	0.2	0.1
Emergency department visits	0.2	0.2	0.1
Days with outpatient services^a^	24.4	31.6	14.0
Days with blood transfusion (any setting)	1.2	1.3	1.0
Days with BTH-related events (any setting)			
Based on symptoms of BTH^b^	4.5	5.2	3.3
Based on treatments for BTH^c^	3.2	4.8	1.2

Abbreviations: BTH, breakthrough hemolysis, HRU, healthcare resource utilization, PNH, paroxysmal nocturnal hemoglobinuria, PPPY, per patient per year.

^a^Intravenous C5 inhibitors (eculizumab and ravulizumab) are administered by a healthcare professional mainly in an outpatient setting. See label-recommended dosing schedule for more details.

^b^Defined as at least 1 of the following conditions: abdominal pain, anemia (excluding aplastic anemia), chronic kidney disease, dysphagia, dyspnea, erectile dysfunction, fatigue, infection, sepsis, respiratory infection, kidney infection, urinary tract infection, pulmonary hypertension, or thrombosis, along with a diagnosis of PNH.

^c^Defined as PNH-related blood transfusion (medical service with diagnosis of PNH and a procedure for blood transfusion) or change in C5 inhibitor dosing schedule (eg, shorter interval, dose escalation).

Among those observed from induction with at least 6 months of follow-up, 46.2% and 11.9% of patients treated with eculizumab and ravulizumab, respectively, required a blood transfusion within the first 6 months post-index. Over a mean ± SD follow-up duration of 25.5 ± 20.6 months, TA was observed in 46.2% of patients treated with eculizumab. TA was observed in 78.2% of patients treated with ravulizumab over a mean ± SD follow-up duration of 20.7 ± 10.3 months.

**Figure 1.**
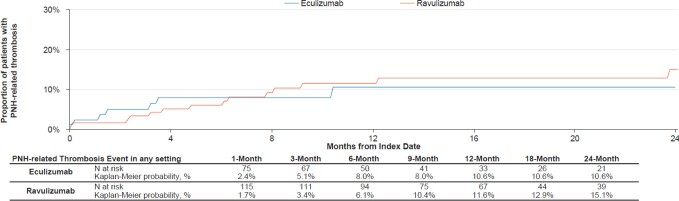
Time to First PNH-Related Thrombosis Event Following Eculizumab or Ravulizumab Initiation Abbreviation: PNH, paroxysmal nocturnal hemoglobinuria.

During a mean ± SD follow-up duration of 17.1 ± 19.6 and 18.4 ± 11.1 months, 12.0% of patients experienced PNH-related thrombosis in the eculizumab and ravulizumab cohorts, of which, 20.0% and 28.6% were observed in an inpatient setting, respectively. Based on the KM analysis, the 6- and 12-month rates of PNH-related thrombosis post-index were 8.0% and 10.6%, respectively for the eculizumab cohort, and 6.1% and 11.6%, respectively, for the ravulizumab cohort (**Figure 1**).

### PNH-Related Direct Healthcare Costs

**Figure 2.**
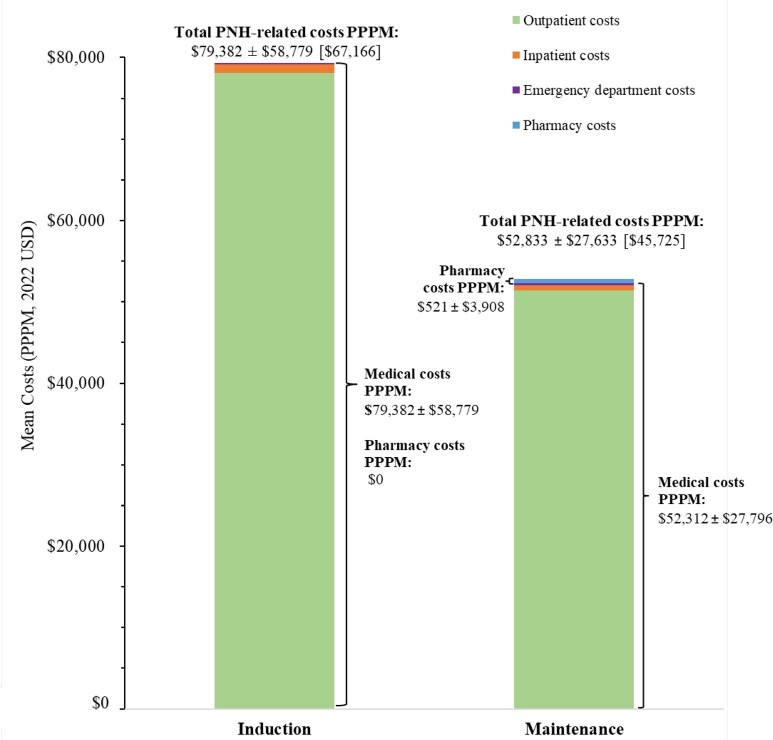
PNH-Related Costs PPPM in Patients With PNH Treated With a C5 Inhibitor Abbreviations: PNH, paroxysmal nocturnal hemoglobinuria; PPPM, per patient per month; USD, US dollars. Notes: 1. Costs PPPM are reported as either mean ± SD or mean ± SD [median]. 2. N = 371 patients treated with a C5 inhibitor (induction phase: n = 175; maintenance phase: n = 362).

**Figure 3.**
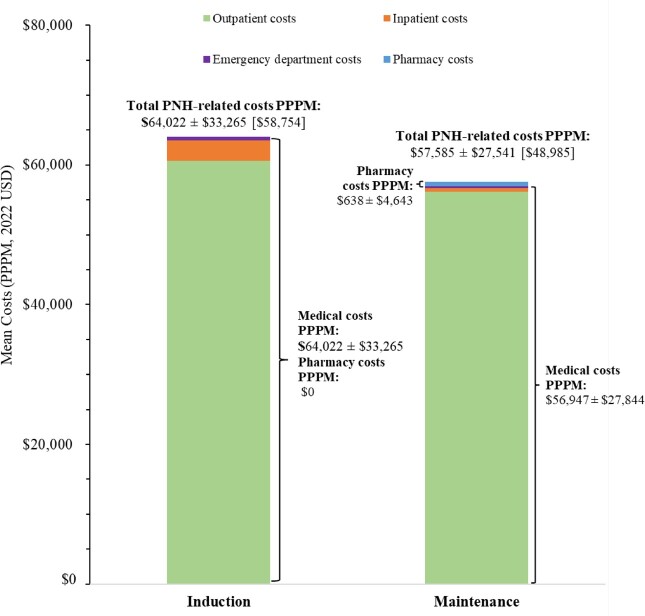
PNH-Related Costs PPPM in Patients With PNH Treated With Eculizumab^1,2^ Abbreviations: PNH, paroxysmal nocturnal hemoglobinuria; PPPM, per patient per month; USD, US dollars. Notes: 1. Costs PPPM are reported as either mean ± SD or mean ± SD [median]. 2. N = 288 patients treated with eculizumab (induction phase: n = 83; maintenance phase: n = 278).

**Figure 4.**
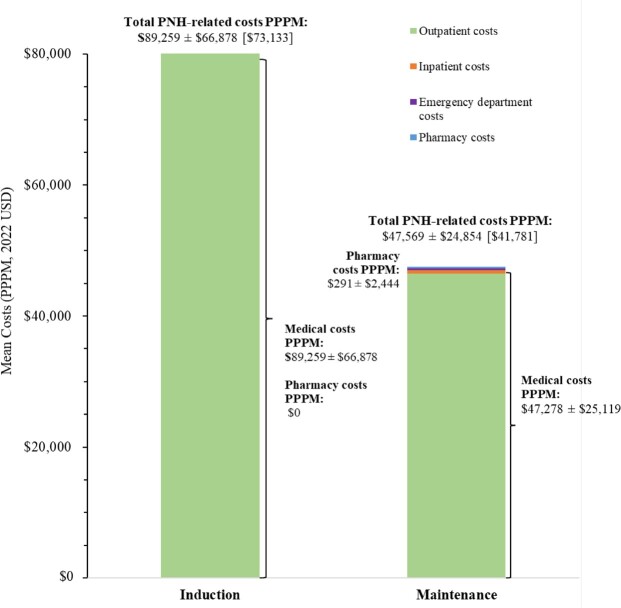
PNH-Related Costs PPPM in Patients With PNH Treated With Ravulizumab Abbreviations: PNH, paroxysmal nocturnal hemoglobinuria; PPPM, per patient per month; USD, US dollars. Notes: 1. Costs PPPM are reported as either mean ± SD or mean ± SD [median]. 2. N = 171 patients treated with ravulizumab (induction phase: n = 117; maintenance phase: n = 171).

Mean PNH-related costs during the induction and maintenance phases for patients treated with a C5 inhibitor, eculizumab, and ravulizumab are presented in **Figure 2**, **Figure 3**, and **Figure 4**, respectively.

Among patients treated with a C5 inhibitor, the mean ± SD (median) cost of blood transfusion was $1483 ± $7148 ($625) per event, while the cost of BTH in any setting was $24 200 ± $35 413 ($19 383) per event when based on symptoms of BTH, and $26 310 ± $23 815 ($26 274) per event when based on treatments for BTH.

The annual total PNH-related costs were estimated at $660 533 PPPY during the first year and $633 984 PPPY for subsequent years among patients treated with C5 inhibitors, of which treatment costs accounted for 94.3% to 94.6% of total PNH-related costs. Among patients treated with eculizumab or ravulizumab, annual total PNH-related costs were estimated at $697 459 PPPY and $612 522 PPPY for the first year, respectively, and $691 022 PPPY and $570 832 PPPY for subsequent years, respectively. Treatment costs accounted for 92.2% of annual total PNH-related costs for patients treated with eculizumab over the entire study period and increased from 96.1% in the first year to 96.4% in subsequent years for patients treated with ravulizumab.

## DISCUSSION

In this large, US claims-based retrospective study, we evaluated the 5-year prevalence of PNH among commercially insured patients and PNH-related HRU and direct costs associated with the C5 inhibitors, eculizumab and ravulizumab, among patients with PNH.

The contemporary 5-year prevalence of PNH (2018-2022) was estimated to be 2.4 per 100 000 persons in commercial claims, and during that period, 30% of patients with a diagnosis of PNH received treatment with a type of complement inhibitor. Due to the lack of specific diagnosis codes for PNH prior to the transition in October 2015 to ICD-10-CM codes used for billing and reporting in healthcare, prevalence rates have been poorly reported and likely underestimated in the US. This study adds to the limited literature on the annual prevalence of PNH, previously reported to be 1.3 per 100 000 persons in 2017. We found that treatment with either eculizumab or ravulizumab in US clinical practice was associated with high PNH-related costs. Over the first year of treatment, total PNH-related costs were estimated to be $697 459 PPPY for treatment with eculizumab and $612 522 PPPY for treatment with ravulizumab, of which treatment costs accounted for 92% and 96% of total PNH-related costs, respectively. Despite receiving treatment with a C5 inhibitor, patients required PNH-related inpatient admissions, PNH-related blood transfusions, and experienced PNH-related thrombosis, suggesting a potential unmet need for more effective treatments among patients with PNH.

This study supports findings from Broderick et al, which used data between January 1, 2018, and December 31, 2020, from Prime Therapeutics and showed that among the 34 and 52 commercially insured patients with PNH who initiated eculizumab and ravulizumab, respectively, the total mean costs in the first year after treatment initiation were $711 785 and $624 911, respectively, of which 80% and 86%, respectively, were due to treatment costs. The costs reported in the current study with a larger sample size, more recent data, and longer follow-up are consistent with these findings. This study also builds on the previous work by Cheng et al, which identified the incremental economic burden associated with transfusion-dependent vs transfusion-free status among patients with PNH who received eculizumab treatment using data between April 1, 2014, and September 30, 2019, from the IBM MarketScan® Research Databases. Specifically, Cheng et al found that patients who were transfusion-dependent incurred $247 848 in excess direct medical costs PPPY relative to patients who were transfusion-free. In the current study, blood transfusions were associated with an average cost of $1483 per event while the average cost of BTH in any setting was $24 200 to $26 310 per event (depending on the definition of a BTH-related event), further quantifying the substantial costs that can be associated with unmet clinical needs among patients with PNH receiving C5 inhibitor treatments.

To expand our understanding of C5 inhibitor treatment outcomes for these patients, the current study also provides a comprehensive real-world assessment of clinically important condition-related HRU among patients with PNH post-treatment with eculizumab or ravulizumab. Findings show that within 12 months of treatment with eculizumab and ravulizumab, respectively, 10.6% and 11.6% of patients experienced PNH-related thrombosis, which is in line with the study by Broderick et al, wherein thrombosis was reported among 14.7% and 13.5% of patients, respectively, at 12 months. Findings of the current study for TA are also in alignment with Broderick et al, where TA was observed in 47.1% and 67.3% of patients in the eculizumab and ravulizumab cohort, respectively, during the 12-month follow-up period. Additionally, in clinical trials, TA at 6 months after treatment initiation was observed in 66.1% to 82.7% of the eculizumab cohort and 73.6% to 87.6% of the ravulizumab cohort, which is consistent with our findings at a similar timepoint when reviewing the proportion of patients who received blood transfusion during that period. By conducting the TA evaluation over an average follow-up period of over 2 years, this study shows that the proportion of patients achieving TA in real-world clinical practice is 46.2% with eculizumab and 78.2% with ravulizumab, suggesting that between one-fifth to half of the patients, depending on the C5 inhibitor, continue to require blood transfusion to maintain disease control over the course of treatment.

### Limitations

This claims-based analysis was subject to several common and inherent limitations. Study findings should only be interpreted in the context of the sample selection criteria. The health plan claims included only diagnosis and procedure codes recorded for reimbursement purposes and may have been subject to coding errors or data omissions. Further, some information including laboratory data, patient weight, patient race, and reasons for diagnosis codes were not available. For blood transfusion data, the units of blood used were not available. Total annual costs associated with eculizumab and ravulizumab use were estimated based on label-recommended dosing schedules, not considering dose adjustments (eg, dose escalation) which may have been needed to achieve disease control, and therefore may have resulted in a cost underestimation. Although the findings of the study indicate associations between the C5 inhibitor treatment received and HRU and costs, causality cannot be inferred. The study sample was restricted to commercially insured patients, with a slightly greater representation from the South and North Central (ie, Midwest) regions, and thus findings may not be generalizable to the entire US population. Finally, further studies are warranted for a comprehensive assessment of real-world outcomes in patients receiving recently approved treatments for PNH.

## CONCLUSIONS

Findings from this study indicated a 5-year PNH prevalence of 2.4 per 100 000 persons in commercial US claims. Despite the association of C5 inhibitor treatment with high annual PNH-related costs, patients with PNH still exhibited BTH and required blood transfusions. Additionally, patients with PNH experienced PNH-related thrombosis, which may indicate a critical unmet need and suggest that patients could benefit from more effective treatment options. The recent US FDA approval of novel treatments for PNH may potentially address these treatment gaps through targeting extravascular hemolysis in addition to intravascular hemolysis, resulting in better disease control.

### Disclosures

S.T. has provided paid consulting services to Novartis Pharmaceuticals Corporation, which funded the development and conduct of this study and manuscript. S.L., J.P., L.G., and G.Y. are employees of Novartis Pharmaceuticals Corporation. M.C. was an employee of Novartis Pharmaceutical Corporation at the time that this study was conducted. D.L.-V., R.D., S.S. and A.G. are employees of Analysis Group, Inc., a consulting company that has provided paid consulting services to Novartis Pharmaceuticals Corporation, which funded the development and conduct of this study and manuscript.

## Supplementary Material

Online Supplementary Material

