# Supplementary material for: Real-World Prevalence and Outcomes of Patients with Paroxysmal Nocturnal Hemoglobinuria Treated with C5 Inhibitors in the US: A Retrospective Claims Database Analysis
Source: J Health Econ Outcomes Res. 2025 Aug 15;12(2):66–74. doi: 10.36469/001c.142049 (PMC12358179; doi:10.36469/001c.142049)
Supplement: Online Supplementary Material [file jheor_2025_12_2_142049_298396.pdf]

Journal of Health Economics and Outcomes Research

Online Supplementary Material

Real-World Prevalence and Outcomes of Patients with Paroxysmal Nocturnal Hemoglobinuria Treated with C5 Inhibitors in the US: A Retrospective Claims Database Analysis. *JHEOR*. 2025;12(2):67-74.
doi:10.36469/jheor.2025.142049

Figure S1: Sample Selection of Patients With PNH

Table S1: Diagnosis Codes for Thrombosis and Breakthrough Hemolysis

This supplementary material has been provided by the authors to give readers additional information about their work.

This is an open-access article distributed under the terms of the Creative Commons Attribution 4.0 International License (CCBY-4.0). View this license's legal deed at http://creativecommons.org/licenses/by/4.0 and legal code at http://creativecommons.org/licenses/by/4.0/legalcode for more information.

Figure S1. Sample Selection of Patients With PNH

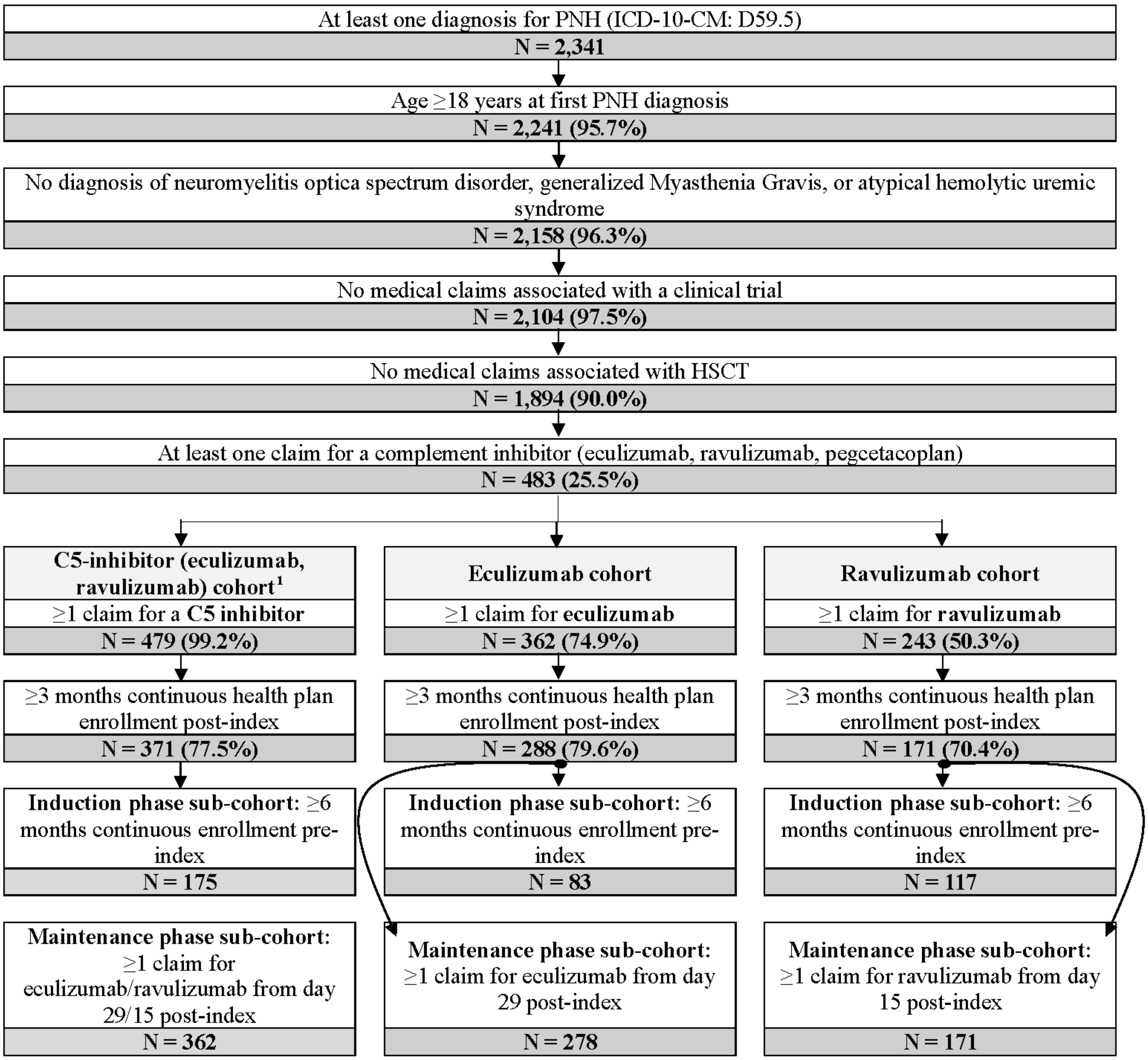

Abbreviations: HSCT, hematopoietic stem cell transplantation; ICD-10-CM, International Classification of Diseases, Tenth Revision, Clinical Modification; PNH, paroxysmal nocturnal hemoglobinuria. N = 371 patients treated with a C5 inhibitor (induction phase: n = 175; maintenance phase: n = 362).

Note: Among the 479 patients included in the C5-inhibitor cohort, a total of 126 were observed with a treatment switch from eculizumab to ravulizumab. These patients were analyzed during their most recent C5 inhibitor treatment, specifically after initiating ravulizumab.

Table S1. Diagnosis Codes for Thrombosis and Breakthrough Hemolysis

**Condition**	**ICD-9-CM**	**ICD-10-CM**
Thrombosis event	325, 415, 416, 43401, 4376, 444, 451, 453, 7454	I80, I81, I82, I26, I27, I74, G08, I636 I676
Breakthrough hemolysis		
Abdominal pain	789.0x, 789.6	R10.0-R10.3, R10.8, R10.9
Anemia (excluding aplastic anemia)	280.x-282.x, 283.0x, 283.1x, 283.9x, 285.x	D50.x-D53.x, D55.x-D58.x, D59.0, D59.1, D59.2, D59.3, D59.4, D59.6, D59.8, D59.9, D60.x, D62.x-D64.x
Chronic kidney disease	403.x, 404.x, 585.x	I12.0, I12.9, I13.0, I13.10, I13.11, I13.2, N18.1-N18.6, N18.9
Dysphagia	787.2x	R13.1.x
Dyspnea	786.02, 786.05	R06.0x
Erectile dysfunction	607.84	N52.0, N52.1
Fatigue	780.71, 780.79	R53.82, R53.83
Kidney and urinary tract infection	590.0, 590.1, 590.80, 590.9, 595, 597.80, 599.0	N10.x, N11.0, N11.1, N12.x, N15.9, N30.x, N34.x, N39.0
Pulmonary hypertension	416.0, 416.8	I27.0, I27.2
Respiratory infection	460-466, 472, 473, 476, 480-488	J00.x-J06.x, J09.x-J18.x, J31.x, J32.x, J37.x, R05.x
Sepsis	038, 790.7, 995.91, 995.92	A40.x, A41.x, R65.2, R78.81

Abbreviations: ICD-9-CM, International Classification of Diseases, Ninth Revision, Clinical Modification; ICD-10-CM, International Classification of Diseases, Tenth Revision, Clinical Modification.
